# Long-term exposure to air pollution and the risk of developing sudden sensorineural hearing loss

**DOI:** 10.1186/s12967-021-03095-8

**Published:** 2021-10-12

**Authors:** Stella Chin-Shaw Tsai, Yi-Chao Hsu, Jung-Nien Lai, Ruey-Hwang Chou, Hueng-Chuen Fan, Frank Cheau-Feng Lin, Ruihong Zhang, Cheng-Li Lin, Kuang-Hsi Chang

**Affiliations:** 1 Department of Medical Research, Tungs’ Taichung Metroharbor Hospital, Taichung, 43503 Taiwan; 2 Department of Otolaryngology, Tungs’ Taichung Metroharbor Hospital, Taichung, 43503 Taiwan; 3 Institute of Biomedical Sciences, Mackay Medical College, New Taipei City, 252 Taiwan; 4 School of Chinese Medicine, College of Chinese Medicine, China Medical University, Taichung, 40402 Taiwan; 5 Department of Chinese Medicine, China Medical University Hospital, Taichung, 40447 Taiwan; 6 Graduate Institute of Biomedical Sciences, China Medical University, Taichung, 40402 Taiwan; 7 Center for Molecular Medicine, China Medical University Hospital, Taichung, 40402 Taiwan; 8 Department of Biotechnology, Asia University, Taichung, 41354 Taiwan; 9 Department of Pediatrics, Department of Medical Research, Tungs’ Taichung Metroharbor Hospital, Taichung, 43503 Taiwan; 10 Department of Rehabilitation, Jen-Teh Junior College of Medicine, Nursing and Management, Miaoli, 35664 Taiwan; 11 Department of Thoracic Surgery, Chung Shan Medical University Hospital, Taichung, 40201 Taiwan; 12 Department of Science and Teaching, The Fourth Central Hospital of Baoding City, Baoding, Hebei China; 13 Management Office for Health Data, China Medical University Hospital, Taichung, 40402 Taiwan; 14 General Education Center, Jen-Teh Junior College of Medicine, Nursing and Management, Miaoli, 35664 Taiwan; 15 Center for General Education, China Medical University, Taichung, 404 Taiwan

**Keywords:** Air pollution, Sudden sensorineural hearing loss (SSNHL), Adjusted hazard ratio (aHR), National Health Insurance program

## Abstract

**Background:**

The association between exposure to air pollution and sudden sensorineural hearing loss (SSNHL) has not been extensively discussed in the literature. Therefore, we conducted this nationwide study to evaluate the risk of SSNHL in Taiwanese residents with exposure to air pollution.

**Methods:**

We enrolled subjects aged older than 20 years with no history of SSNHL from 1998 to 2010, and followed up until developing SSNHL, withdrawn from the National Health Insurance program, and the end of the database (2011/12/31). The air quality data are managed by Taiwan Environmental Protection Administration. The annual concentrations of PM_2.5_, SO_2_, CO, NO, and NO_2_ from 1998 to 2010 were classified into the three levels according to tertiles. We calculated the annual average of pollutants from baseline until the end of the study, and classified into tertiles. The adjusted hazard ratio (aHR) was estimated by using the multivariate Cox proportional hazard model.

**Results:**

When considered continuous air pollutants concentration, subjects who exposed with higher concentration of CO (aHR = 2.16, 95% CI 1.50–3.11), NO (aHR = 1.02, 95% CI 1.01–1.03), and NO_2_ (aHR = 1.02, 95% CI 1.01–1.04) developing significant higher risk of SSNHL. When classified air pollutants concentration into low, moderate and high level by tertiles, and selected low level as reference, patients exposed with moderate (aHR = 1.56, 95% CI 1.20–2.04) or high level (aHR = 1.33, 95% CI 1.01–1.75) of PM_2.5_ showed significant higher risk of developing SSNHL.

**Conclusion:**

This study indicated an increased risk of SSNHL in residents with long-term exposure to air pollution. Nevertheless, further experimental, and clinical studies are needed to validate the study findings.

**Supplementary Information:**

The online version contains supplementary material available at 10.1186/s12967-021-03095-8.

## Introduction

Air pollution has become an important environmental issue in the last decade, especially in the developing and developed countries. The levels of air pollutants are highly and positively correlated with population density, vehicle emissions, agriculture, industrial emissions, power plants, and fossil fuel combustion [1, 2]. Exposure to air pollutants triggers systemic and tissue-specific inflammation [3, 4]. Previous studies have indicated that exposure to air pollution increases the risks of degeneration diseases, cerebrovascular and cardiovascular diseases, immunological diseases, malignant tumors, and ophthalmological diseases [5–12]. In addition, air pollution is the major environment-related risk factor for human mortality [13].

Although viral infection, environmental or occupational factors (such as loud noises, heavy metals, and organic solvents), autoimmune diseases, cardiovascular diseases, accidental events, endothelial dysfunction, metabolic diseases, and health habits (such as smoking and alcohol consumption)are risk factors for sudden deafness (sudden sensorineural hearing loss, SSNHL), the complex etiology of SSNHL remains unclear [14–23]. Exposure to air pollution increases oxidative stress, which can play an important role in endothelial dysfunction [24]. A previous study reports air pollution as a risk factor of developing sensorineural hearing loss [25]. However, the association between exposure to air pollution and SSNHL has not been extensively discussed in the literature. Therefore, we conducted this nationwide study to evaluate the risk of SSNHL in Taiwanese residents with exposure to air pollution.

## Methods

### Data source and study subjects

Taiwan government built a nationwide database, named National Health Insurance Database (NHIRD), since 1995 and included the medical record of health insurance single payer in Taiwan. The medical record included the history of outpatients, hospitalization, the prescriptions of medications and other medical services. As of today, more than 99% of Taiwan population were enrolled in the database. We conducted this study by Longitudinal Health Insurance Database (LHID 2000), which was randomly selected 1 million study subjects from NHIRD. All identification number were encrypted for the patients’ privacy. The history diagnoses are coded according to the International Classification of Disease, Ninth Revision, Clinical Modification (ICD-9-CM). The Research Ethics Committee of China Medical University and Hospital in Taiwan approved the study (CMUH-104-REC2-115-R4).

We enrolled subjects aged older than 20 years with no history of SSNHL from 1998 to 2010, and followed up until developing SSNHL, withdrawn from the NHI program, and the end of the database (2011/12/31).

### Exposure measurement

The data regarding the air pollutants were collected from 74 ambient air quality monitoring stations across Taiwan. The air quality data are managed by Taiwan Environmental Protection Administration. The annual concentrations of PM_2.5_, SO_2_, CO, NO, and NO_2_ from 1998 to 2010 were classified into the three levels according to tertiles: the PM_2.5_ concentrations of the low-, mid-, and high-level groups were < 30.29 (μg/m^3^), 30.29–37.61 (μg/m^3^) and > 37.61 (μg/m^3^), respectively. The SO_2_ concentrations of the low-, mid-, and high-level groups were < 3.57 (ppb), 3.57–5.51 (ppb) and > 5.51 (ppb), respectively. The CO concentrations of the low-, mid-, and high-level groups were < 0.61 (ppm), 0.61–0.76 (ppm) and > 0.76 (ppm) respectively. The NO concentrations of the low-, mid-, and high-level groups were < 5.04 (ppb), 5.04–8.90 (ppb) and > 8.90 (ppb), respectively. The NO_2_ concentrations of the low-, mid-, and high-level groups were < 19.48 (ppb), 19.48–25.55 (ppb) and > 25.55 (ppb), respectively.

### Main outcome and covariates

The main outcome of this study was the SSNHL (ICD-9-CM: 3882; ICD-10-CM: H91.20, H91.21, H91.22, H91.23). SSNHL is most defined as sensorineural hearing loss of 30 dB or greater over at least three contiguous audiometric frequencies occurring within a 72-h period. This definition must be confirmed with pure tone audiometry and history taking before insurance could pay for the appropriate treatment. The demographic factors we considered included age, insurance fee, urbanization, and comorbidities. The common comorbidities including hypertension (HT, ICD-9-CM codes 401–405), diabetes mellitus (DM, ICD-9-CM code250), stroke, head injury (ICD-9-CM codes 850–854), chronic kidney disease (CKD, ICD-9-CM code 585), ischemic heart disease (IHD, ICD-9-CM codes 410–414), alcoholism (ICD-9-CM codes 305.0 and 303), asthma (ICD-9-CM code 493), Chronic obstructive pulmonary disease (COPD, ICD-9-CM codes 490–492, 494, and 496), impacted cerumen (IC, ICD-9-CM code 380.4), suppurative and unspecified otitis media (SUOM, ICD-9-CM codes 382.0, 382.1, 382.2, 382.3, 382.4 and 382.9), chronic serous otitis media (CSOM, ICD-9-CM codes 381.10 and 381.19), otosclerosis (ICD-9-CM code 387.9) and rheumatoid arthritis (RA, ICD-9-CM code 714) were presented as confounding factors in this study.

### Statistical analysis

We presented continuous variables by mean and standard deviation; categorical variables were shown by number and percentage. The difference between with and without SSNHL were tested by t-test and chi-square test for continuous and categorical variable, respectively. To analyze the exposures across the long-term period, we calculated the annual average of pollutants from baseline until the end of the study, and classified into tertiles: the low, moderate, and high-level groups. When compared mean and classified pollutants concentration in four level of urbanization (highly, moderately, boomtown and others), ANOVA test and chi-square test was applied, respectively.

The incidence rates of SSNHL were calculated, and the hazard ratio (HR) was estimated by using the multivariate Cox proportional hazard model, adjusting for age, sex, insurance fee, urbanization, and comorbidities.

## Results

We totally enrolled 64,321 subjects in this study. 353 with SSNHL and the other 63,968 without SSNHL. Table 1 presented the distribution of demographics and comorbidities between two groups. The mean age of SSNHL and non-SSNHL were 45.58 and 39.12 years, and with 8.47 and 11.71 follow up years, respectively. Patients with SSNHL had significant higher percentage of HT (45.6%), DM (17.3%), IHD (27.5%), IC (12.5%), SUOM (11.6%) and COPD (29.5%) than non-SSNHL group. The distribution of the levels of insurance fee showed insignificant between two groups. Most study subjects lived in highly (34.3%) and moderately (32.6%) urbanized area. Table 2 showed the distribution of different pollutants concentration and SSNHL. SO_2_ and NO_2_ concentration showed insignificant difference between SSNHL and non-SSNHL group when calculated by mean or classified into levels. The mean of CO (0.76 vs 0.72) and NO (12.6 vs 11.0) concentration was significant higher in the group of SSNHL, respectively. Table 3 showed the association between pollutants concentration and urbanized level. The level of CO, NO and NO_2_ showed the mean 0.81, 0.69, 0.70 and 0.59 (ppm); 14.14, 9.94, 10.73 and 6.80 (ppb); 24.58, 22.06, 23.39, and 18.63 (ppb) from highly urbanized, moderately, boomtown to others, respectively. The pollutants we mentioned above might highly associated with the level of urbanization. The risk of SSNHL and the level of air pollutants were calculated in Table 4. When considered continuous air pollutants concentration, subjects who exposed with higher concentration of CO (adjusted hazard ratio (aHR) = 2.16, 95% CI 1.50–3.11), NO (aHR = 1.02, 95% CI 1.01–1.03), and NO_2_ (aHR = 1.02, 95% CI 1.01–1.04) developing significant higher risk of SSNHL. When classified air pollutants concentration into low, moderate, and high level by tertiles, and selected low level as reference, patients exposed with moderate (aHR = 1.56, 95% CI 1.20–2.04) or high level (aHR = 1.33, 95% CI 1.01–1.75) of PM_2.5_ showed significant higher risk of developing SSNHL.

**Table 1:** Distribution of the demographic data and comorbidities of the study participants

Covariates	SSNHL (n = 353)		Non- SSNHL (n = 63,968)		p	Total (n = 64,321)	
Age							
Mean (SD)	45.58 (15.36)		39.12 (14.99)		< 0.001	39.16 (15.00)	
Follow years							
Mean (SD)	8.47 (2.49)		11.71 (0.91)		< 0.001	11.69 (0.96)	
HT	161	45.6%	19,464	30.4%	< 0.001	19,625	30.5
DM	61	17.3%	7023	11.0%	< 0.001	7084	11.0
Stroke	19	5.4%	2586	4.0%	0.255	2605	4.0
Head injury	27	7.6%	5237	8.2%	0.787	5264	8.2
CKD	10	2.8%	1584	2.5%	0.796	1594	2.5
IHD	97	27.5%	10,776	16.8%	< 0.001	10,873	16.9
Alcoholism	7	2.0%	975	1.5%	0.629	982	1.5
Nicotine	5	1.4%	1814	2.8%	0.149	1819	2.8
Asthma	50	14.2%	7581	11.9%	0.208	7631	11.9
COPD	104	29.5%	13,997	21.9%	0.001	14,101	21.9
RA	1	0.3%	191	0.3%	1.000	192	0.3
IC	44	12.5%	3466	5.4%	< 0.001	3510	5.5%
SUOM	41	11.6%	3651	5.7%	< 0.001	3692	5.7%
CSOM	2	0.6%	196	0.3%	0.296	198	0.3%
Otosclerosis	0	0	14	0.02%	1.000	14	0.02%
Insurance fee							
Lowest	59	16.7%	10,633	16.6%	0.316	10,692	16.6
2nd	102	28.9%	20,987	32.8%		21,089	32.8
3rd	91	25.8%	14,280	22.3%		14,371	22.3
Highest	101	28.6%	18,068	28.2%		18,169	28.2
Urbanization							
Highly	108	30.6%	21,946	34.3%	0.002	22,054	34.3
Moderately	138	39.1%	20,837	32.6%		20,975	32.6
Boomtown	40	11.3%	10,856	17.0%		10,896	16.9
Others	67	19.0%	10,329	16.1%		10,396	16.2

HT: hypertension; DM: diabetes mellitus; CKD: chronic kidney disease; IHD: ischemic heart disease; Nicotine: nicotine dependence; COPD: chronic obstructive pulmonary disease; RA: rheumatoid arthritis; IC: impacted cerumen; SUOM: suppurative and unspecified otitis media; CSOM: chronic serous otitis media

**Table 2:** Distribution of air pollutant exposure in study participants

Pollutants	Levels	SSNHL (n = 353)		Non- SSNHL (n = 63,968)		p	Total (n = 64,321)	
PM_2.5_ (μg/m^3^)	Mean (SD)	35.07 (8.74)		34.79 (8.75)		0.556	34.79 (8.75)	
	Low	92	26.1%	21,393	33.4%	0.007	21,485	33.4
	Moderate	133	37.7%	20,202	31.6%		20,335	31.6
	High	128	36.3%	22,373	35.0%		22,501	35.0
SO_2_ (ppb)	Mean (SD)	4.91 (2.50)		4.98 (2.41)		0.557	4.98 (2.41)	
	Low	127	36.0%	21,641	33.8%	0.519	21,768	33.8
	Moderate	111	31.4%	19,691	30.8%		19,802	30.8
	High	115	32.6%	22,636	35.4%		22,751	35.4
CO (ppm)	Mean (SD)	0.76 (0.33)		0.72 (0.27)		0.010	0.72 (0.27)	
	Low	124	35.1%	22,582	35.3%	0.204	22,706	35.3
	Moderate	93	26.3%	19,242	30.1%		19,335	30.1
	High	136	38.5%	22,144	34.6%		22,280	34.6
NO (ppb)	Mean (SD)	12.60 (12.70)		11.00 (10.13)		< 0.001	11.01 (10.15)	
	Low	119	33.7%	21,754	34.0%	0.936	21,873	34.0
	Moderate	109	30.9%	20,140	31.5%		20,249	31.5
	High	125	35.4%	22,074	34.5%		22,199	34.5
NO_2_ (ppb)	Mean (SD)	22.99 (7.49)		22.56 (6.55)		0.216	22.56 (6.56)	
	Low	114	32.3%	20,002	31.3%	0.156	20,116	31.3
	Moderate	101	28.6%	21,284	33.3%		21,385	33.2
	High	138	39.1%	22,682	35.5%		22,820	35.5

SD: standard deviation; ppb: parts per billion; ppm: parts per million

**Table 3:** Distributions of air pollutants among urbanization zones

Pollutants	Levels	Highly Urbanized (n = 22,054)		Moderately Urbanized (n = 22,975)		Boomtown (n = 10,896)		Others (n = 10,396)		p	Total (n = 64,321)	
PM_2.5_ (μg/m^3^)	Mean (SD)	32.71 (7.85)		35.68 (9.16)		37.24 (8.44)		34.86 (9.04)		< 0.001	34.79 (8.75)	
	Low	9906	44.9	6335	30.2	2651	24.3	2593	24.9	< 0.001	21,485	33.4
	Moderate	7680	34.8	6067	28.9	3538	32.5	3050	29.3		20,335	31.6
	High	4468	20.3	8573	40.9	4707	43.2	4753	45.7		22,501	35.0
SO_2_ (ppb)	Mean (SD)	4.88 (2.09)		5.23 (2.53)		5.76 (2.74)		3.86 (1.97)		< 0.001	4.98 (2.41)	
	Low	7275	33.0	6100	29.1	2700	24.8	5693	54.8	< 0.001	21,768	33.8
	Moderate	6833	31.0	6737	32.1	3233	29.7	2999	28.8		19,802	30.8
	High	7946	36.0	8138	38.8	4963	45.5	1704	16.4		22,751	35.4
CO (ppm)	Mean (SD)	0.81 (0.30)		0.69 (0.24)		0.70 (0.22)		0.59 (0.21)		< 0.001	0.72 (0.27)	
	Low	5516	25.0	7839	37.4	3492	32.0	5859	56.4	< 0.001	22,706	35.3
	Moderate	4810	21.8	6904	32.9	4534	41.6	3087	29.7		19,335	30.1
	High	11,728	53.2	6232	29.7	2870	26.3	1450	13.9		22,280	34.6
NO (ppb)	Mean (SD)	14.14 (12.66)		9.94 (8.21)		10.73 (8.43)		6.80 (6.75)		< 0.001	11.01 (10.15)	
	Low	4156	18.8	7342	35.0	3177	29.2	7198	69.2	< 0.001	21,873	34.0
	Moderate	6556	29.7	7551	36.0	4205	38.6	1937	18.6		20,249	31.5
	High	11,342	51.4	6082	29.0	3514	32.3	1261	12.1		22,199	34.5
NO_2_ (ppb)	Mean (SD)	24.58 (6.51)		22.06 (6.30)		23.39 (5.19)		18.43 (6.41)		< 0.001	22.56 (6.56)	
	Low	4245	19.2	7283	34.7	2547	23.4	6041	58.1	< 0.001	20,116	31.3
	Moderate	6127	27.8	7354	35.1	4930	45.2	2974	28.6		21,385	33.2
	High	11,682	53.0	6338	30.2	3419	31.4	1381	13.3		22,820	35.5

ppb: parts per billion; ppm: parts per million

**Table 4:** Adjusted HR of SSNHL in the moderate and high concentration groups compared to the values in the low concentration group

Pollutants	Levels	n of SSNHL	Person-Years	IR	aHR	95%CI		p
PM_2.5_ (μg/m^3^)	Continuous				1.01	0.99	1.02	0.311
	Low	92	251,573	0.37	Reference			
	Moderate	133	238,157	0.56	1.56	1.20	2.04	0.001
	High	128	262,340	0.49	1.33	1.01	1.75	0.043
SO_2_ (ppb)	Continuous				1.01	0.96	1.05	0.811
	Low	127	253,994	0.50	Reference			
	Moderate	111	231,810	0.48	1.01	0.78	1.31	0.927
	High	115	266,267	0.43	0.96	0.74	1.24	0.754
CO (ppm)	Continuous				2.16	1.50	3.11	< 0.001
	Low	124	265,999	0.47	Reference			
	Moderate	93	226,689	0.41	0.96	0.73	1.26	0.754
	High	136	259,382	0.52	1.27	0.99	1.65	0.065
NO (ppb)	Continuous				1.02	1.01	1.03	< 0.001
	Low	119	255,830	0.47	Reference			
	Moderate	109	237,175	0.46	1.14	0.86	1.49	0.363
	High	125	259,066	0.48	1.22	0.93	1.61	0.151
NO_2_ (ppb)	Continuous				1.02	1.01	1.04	0.012
	Low	114	235,391	0.48	Reference			
	Moderate	101	250,758	0.40	0.93	0.71	1.23	0.625
	High	138	265,922	0.52	1.25	0.96	1.63	0.105

n of SSNHL: number of patients with hearing loss; IR: incidence rate (per 1000 person-years); IR: incidence rate; aHR: adjusted hazard ratio in the multivariate analysis after adjusting for age, insurance fee, urbanization, HT, DM, stroke, head injury, CKD, IHD, alcoholism, nicotine dependence, COPD, asthma, RA, impacted cerumen, suppurative and unspecified otitis media, chronic serous otitis media, and otosclerosis

## Discussion

This retrospective cohort study combined two large, longitudinal databases to evaluate the risk of SSNHL in Taiwanese residents with chronic exposure to air pollution. During the approximately 11-year follow-up, we enrolled 64,321 residents (353 in SSNHL; 63,968 in non-SSNHL) and found the participants who were exposed to PM_2.5_, CO, NO, and NO_2_had a significantly higher risk of SSNHL. However, SO_2_ exposure was not similarly correlated.

The association between exposure to air pollution and development of hearing loss is unclear. In 2019, a nested case–control indicated that short-term exposure to NO_2_ significantly increased the risk of SSNHL (adjusted odds ratio: 3.12 [95% confidence interval: 2.16–4.49)] [26]. Another large scale study in Korea found a weak relationship between daily numbers of SSNHL patients and PM levels [27]. Nevertheless, the association between long-term exposure to air pollution and development of SSNHL remains debatable and requires further clarification.

According to Table 3, the distributions of PM_2.5_ and SO_2_ were not consistent with urbanization levels. This discrepancy may result from intensive agricultural activities in the less urbanized cities [28–30]. Fossil fuel combustion in industrial facilities or power plants is the major source of SO_2_ emissions [31]. Because of the high land value and appropriate land and emission standards, industrial factories or power plants are not preferably setup in areas with a high population density.

This nationwide study with minimized selection bias has several limitations. First, we considered the medical convenience; thus, the definition of residential address was based on the location of medical institutions where participants most frequently received therapy for acute respiratory infections. According to this definition, there was a potential bias of excluding subjects without related medical records. However, evidence indicates that these people most likely had less air pollutant exposure [32–34]. This may result in an underestimation of SSNHL cases. Second, SSNHL is an emergency otologic condition. There were more frequent hospital visits by residents in highly urbanized cities with high levels of air pollutants than other areas. Although this may result in surveillance bias and an overestimation of the risk of SSNHL, previous evidence indicates the obvious narrowing of health disparities between urban and rural areas because of the NHI program removing some barriers and providing free health care in the less urbanized areas [35, 36]. Third, although the records of SSNHL were acquired according to the claim data from the NHIRD instead of by physical examination, the SSNHL diagnosis was validated by audiology examinations and neurological findings to avoid strict fines from Taiwan Bureau of National Health Insurance. Fourth, patients’ occupation and health behaviors, such as smoking and alcohol consumption, which are considered risk factors of SSNHL, were not available from the NHIRD. Hence, we considered insurance fees, COPD, asthma, nicotine dependence, and alcoholism in the multivariate analysis. Smoking behavior was highly correlated with the development of COPD and asthma [37–40]. The diagnosis of alcoholism was according to patients' attitudes and drinking behaviors [41]. In several previous NHIRD-related studies, COPD, asthma, nicotine dependence, and alcoholism were considered risk factors instead of smoking and drinking [42–44]. Fifth, traffic-related air pollutants co-occur with noise. It is not feasible to clarify the contributions of air pollution and noise individually due to the lack of noise data in the two large databases. Therefore, the application of the present study is limited. Despite these limitations, the present nationwide study with a long follow-up period might reduce the impacts of biases. We divided the five pollutants into high and low by median, and combined PM_2.5_ with any of other four pollutants to evaluate the risk of SSNHL (Additional file 1). However, it seems the synergistic effects are not obvious.

## Conclusion

In conclusion, we redefined the residential area by the location of hospital or clinics rather than the addresses of group insurance applicants and considered the proxy covariates of health behaviors to overcome the inherent limitation of the NHIRD. This study indicated an increased risk of SSNHL in residents with exposure to air pollution. Nevertheless, further experimental, and clinical studies are needed to validate the study findings.

## Supplementary Information

**Additional file 1.** Combined PM_2.5_ with any of other four pollutants to evaluate the risk of SSNHL.

## Data Availability

Data are available from the NHIRD published by Taiwan National Health Insurance Bureau. Due to the ‘Personal Information Protection Act’, data cannot be made publicly available (http://nhird.nhri.org.tw/en/index.html).
