# Supplementary material for: Long-term exposure to air pollution and the risk of developing sudden sensorineural hearing loss
Source: J Transl Med. 2021 Oct 12;19:424. doi: 10.1186/s12967-021-03095-8 (PMC8507317; doi:10.1186/s12967-021-03095-8)
Supplement: Supplementary file 1 — Additional file 1. Combined PM2.5 with any of other four pollutants to evaluate the risk of SSNHL. [file 12967_2021_3095_MOESM1_ESM.docx]

Supplementary table.

|  | | aHR | 95%CI | | p |
| --- | --- | --- | --- | --- | --- |
| PM_2.5_ | <median | Reference |  |  |  |
|  | ≥median | 1.37 | 1.11 | 1.70 | 0.004 |
| SO_2_ | <median | Reference |  |  |  |
|  | ≥median | 1.06 | 0.86 | 1.31 | 0.597 |
| CO | <median | Reference |  |  |  |
|  | ≥median | 1.35 | 1.09 | 1.68 | 0.007 |
| NO | <median | Reference |  |  |  |
|  | ≥median | 1.34 | 1.07 | 1.68 | 0.010 |
| NO_2_ | <median | Reference |  |  |  |
|  | ≥median | 1.29 | 1.03 | 1.61 | 0.027 |
| PM_2.5_ <median & SO_2_ <median | | Reference |  |  |  |
| PM_2.5_ <median & SO_2_ ≥median | | 0.98 | 0.71 | 1.36 | 0.913 |
| PM_2.5_ ≥median & SO_2_ <median | | 1.35 | 1.00 | 1.83 | 0.052 |
| PM_2.5_ ≥median & SO_2_ ≥median | | 1.37 | 1.04 | 1.81 | 0.027 |
| PM_2.5_ <median & CO <median | | Reference |  |  |  |
| PM_2.5_ <median & CO ≥median | | 1.14 | 0.82 | 1.58 | 0.426 |
| PM_2.5_ ≥median & CO <median | | 1.17 | 0.86 | 1.60 | 0.313 |
| PM_2.5_ ≥median & CO ≥median | | 1.76 | 1.31 | 2.36 | <0.001 |
| PM_2.5_ <median & NO <median | | Reference |  |  |  |
| PM_2.5_ <median & NO ≥median | | 0.96 | 0.69 | 1.35 | 0.829 |
| PM_2.5_ ≥median & NO <median | | 1.02 | 0.75 | 1.39 | 0.902 |
| PM_2.5_ ≥median & NO ≥median | | 1.64 | 1.24 | 2.16 | <0.001 |
| PM_2.5_ <median & NO_2_ <median | | Reference |  |  |  |
| PM_2.5_ <median & NO_2_ ≥median | | 1.11 | 0.80 | 1.54 | 0.523 |
| PM_2.5_ ≥median & NO_2_ <median | | 1.21 | 0.89 | 1.64 | 0.231 |
| PM_2.5_ ≥median & NO_2_ ≥median | | 1.74 | 1.29 | 2.37 | <0.001 |

aHR: adjusted hazard ratio in the multivariate analysis after adjusting for age, insurance fee, urbanization, HT, DM, stroke, head injury, CKD, IHD, alcoholism, nicotine dependence, COPD, asthma, RA, impacted cerumen, suppurative and unspecified otitis media, chronic serous otitis media, and otosclerosis.
